# *Acinetobacter baumannii* Isolates from COVID-19 Patients in a Hospital Intensive Care Unit: Molecular Typing and Risk Factors

**DOI:** 10.3390/microorganisms10040722

**Published:** 2022-03-28

**Authors:** Mariateresa Ceparano, Valentina Baccolini, Giuseppe Migliara, Claudia Isonne, Erika Renzi, Daniela Tufi, Corrado De Vito, Maria De Giusti, Maria Trancassini, Francesco Alessandri, Giancarlo Ceccarelli, Francesco Pugliese, Paolo Villari, Maria Angiulli, Stefania Battellito, Arianna Bellini, Andrea Bongiovanni, Lucilla Caivano, Marta Castellani, Monica Coletti, Alessia Cottarelli, Ludovica D’Agostino, Andrea De Giorgi, Chiara De Marchi, Irma Germani, Dara Giannini, Elisa Mazzeo, Shadi Orlandi, Matteo Piattoli, Eleonora Ricci, Leonardo Maria Siena, Alessandro Territo, Gianluca Vrenna, Stefano Zanni, Carolina Marzuillo

**Affiliations:** 1 Department of Public Health and Infectious Diseases, Sapienza University of Rome, 00185 Rome, Italy; valentina.baccolini@uniroma1.it (V.B.); giuseppe.migliara@uniroma1.it (G.M.); claudia.isonne@uniroma1.it (C.I.); erika.renzi@uniroma1.it (E.R.); daniela.tufi@uniroma1.it (D.T.); corrado.devito@uniroma1.it (C.D.V.); maria.degiusti@uniroma1.it (M.D.G.); maria.trancassini@uniroma1.it (M.T.); giancarlo.ceccarelli@uniroma1.it (G.C.); paolo.villari@uniroma1.it (P.V.); angiulli.1845548@studenti.uniroma1.it (M.A.); stefania.battellito@uniroma1.it (S.B.); arianna.bellini@uniroma1.it (A.B.); andrea.bongiovanni@uniroma1.it (A.B.); marta.castellani@uniroma1.it (M.C.); monica.coletti@uniroma1.it (M.C.); alessia.cottarelli@uniroma1.it (A.C.); ludovica.dagostino@uniroma1.it (L.D.); andrea.degiorgi@uniroma1.it (A.D.G.); chiara.demarchi@uniroma1.it (C.D.M.); dara.giannini@uniroma1.it (D.G.); elisa.mazzeo@uniroma1.it (E.M.); shadi.orlandi@uniroma1.it (S.O.); eleonora.ricci@uniroma1.it (E.R.); leonardo.siena@uniroma1.it (L.M.S.); alessandro.territo@uniroma1.it (A.T.); stefano.zanni@uniroma1.it (S.Z.); carolina.marzuillo@uniroma1.it (C.M.); 2 Microbiology and Virology Unit, Umberto I Teaching Hospital, Sapienza University of Rome, 00161 Rome, Italy; 3 Department of Anaesthesia and Intensive Care Medicine, Umberto I Teaching Hospital, Sapienza University of Rome, 00161 Rome, Italy; francesco.alessandri@uniroma1.it (F.A.); f.pugliese@uniroma1.it (F.P.); irmager@libero.it (I.G.); matteo.piattoli@uniroma1.it (M.P.); 4 Department of General and Specialist Surgery “P. Stefanini”, Sapienza University of Rome, 00185 Rome, Italy; 5 Department of Molecular Medicine, Sapienza University of Rome, 00185 Rome, Italy; lucilla.caivano@uniroma1.it (L.C.); gianluca.vrenna@uniroma1.it (G.V.)

**Keywords:** *Acinetobacter baumannii*, COVID-19, intensive care unit, molecular typing

## Abstract

Infections caused by *Acinetobacter baumannii* represent a major concern for intensive care unit (ICU) patients. However, the epidemiology of these infections among COVID-19 patients has not been fully explored. The aims of this study were (i) to characterize the clonal spread of *A. baumannii* among COVID-19 patients admitted to the ICU of the Umberto I hospital of Rome during the first year of the pandemic and (ii) to identify risk factors for its acquisition. Isolates were analysed by pulsed-field gel electrophoresis, and a multivariable regression model was constructed. Adjusted odds ratios (aORs) and 95% confidence intervals (CIs) were calculated. Overall, 193 patients were included, and 102 strains were analysed. All isolates had highly antibiotic-resistant profiles and derived from two genotypes. The cumulative incidence of *A. baumannii* acquisition (colonization or infection) was 36.8%. Patients with *A. baumannii* had higher mortality and length of stay. Multivariable analysis showed that previous carbapenem use was the only risk factor associated with *A. baumannii* acquisition (aOR: 4.15, 95% CI: 1.78–9.64). We documented substantial *A. baumannii* infections and colonization and high levels of clonal transmission. Given the limited treatment options, effective prevention and containment strategies to limit the spread of *A. baumannii* should be implemented.

## 1. Introduction

*Acinetobacter baumannii* is a Gram-negative bacterium that belongs to the Moraxellaceae family [1]. It can survive for long periods on surfaces, including dry surfaces and human skin, which can facilitate its persistence and spread in healthcare facilities [2]. In addition, extensive antibiotic abuse and poor stewardship have contributed to an increase in multidrug-resistant (MDR) strains of this pathogen [3], which have a marked tendency to develop multiple resistance mechanisms, resulting in problematic antimicrobial management [4]. For these reasons, infections caused by MDR *A. baumannii* represent a major concern for patients admitted to intensive care units (ICU) [5], where inappropriate therapy and limited therapeutic options contribute to the increased mortality and morbidity rates registered in infected patients [6].

Since March 2020, sustained transmission of SARS-CoV-2 has resulted in high rates of ICU admission all over the world, including Italy [7]. Within this context, the primary focus of mitigating the spread of SARS-CoV-2 may have inadvertently diverted attention away from traditional healthcare-associated infection (HAI) prevention programs [8]. Indeed, many healthcare facilities have had to contend with physical space limitations, constrained availability of personnel, shortages in personal protective equipment, and large numbers of patients [9], leading to disruptions in surveillance efforts, process measures, and containment strategies [8]. These factors, combined with healthcare workers’ fear of becoming infected and patients’ long length of stay, may have favoured cross-contamination of microorganisms between patients that, given their frequently critical conditions, may then have led to subsequent bacterial coinfections [10].

Worldwide, several studies have reported high incidence of infections due to methicillin-resistant *S. aureus*, carbapenem-resistant *A. baumannii*, carbapenem-resistant *Enterobacteriaceae*, and *C. auris* among COVID-19 patients admitted to ICUs [11]. In Italy, a recent report found that one of the most commonly isolated microorganisms in COVID-19 patients was carbapenem-resistant *A. baumannii*, suggesting a worsening in its ICU prevalence likely due to the occurrence of the pandemic [12]. However, the origin and specific nature of these coinfections have yet to be fully explored [8]. The aim of this study was twofold: (i) to describe and quantify the clonal transmission of *A. baumannii* among COVID-19 patients hospitalized in the ICU of Umberto I teaching hospital of Rome during the first year of the pandemic and (ii) to identify risk factors for its acquisition, which should improve our understanding of the epidemiology of these coinfections and provide evidence to physicians to support the implementation of prevention strategies.

## 2. Materials and Methods

This study consisted of two parts: (i) we used microbiological methods to identify any clonality between *A. baumannii* isolates collected from the SARS-CoV-2 patients admitted to the ICU from 1 March 2020 to 28 February 2021 (follow-up terminated on 31 March 2021); (ii) we investigated risk factors for *A. baumannii* acquisition (i.e., colonization or infection), with data on the above patients being retrospectively reviewed.

### 2.1. Molecular Typing

The identification and antimicrobial susceptibility of the strains of *A. baumannii* obtained from clinical specimens were studied by an automatized Vitek 2 system (Biomerieux, Marcy-l’Étoile, France). The strains were collected, isolated as pure cultures, and stored at −80 °C with glycerol for genotypic typing. For each patient, if repeated *A. baumannii* isolates were obtained over time from the same type of clinical specimen, only the first was considered and subsequently analysed by pulsed-field gel electrophoresis (PFGE). A 10 mL culture grown overnight with shaking in brain–heart infusion broth at 37 °C was pelleted, washed in 500 μL EET buffer (100 mmol/L EDTA, 10 mmol/L EGTA, 10 mmol/L Tris pH 8.0), and resuspended in 200 μL of the same buffer. This cell suspension was embedded into plugs of low-melting-point agarose. The cells were lysed by incubation of the disks at 50 °C for 24 h in a solution of proteinase K (1 mg/mL) and sodium dodecyl sulfate (1%) in EET buffer. This was followed by 5 washes in 14 mL of TE buffer (10 mmol/L Tris, 1 mmol/L EDTA pH 7.5) for 1 h with gentle agitation. The genomic DNA was digested with Apa I (New England Biolabs, Hitchin Herts, UK). The plugs were embedded in 1% agarose gel wells. Subsequently, the separation of the DNA bands was carried out using a CHEF DR II system (BioRad, Hercules, CA, USA) at 6 V/cm^2^ for 20 h at 14 °C, and the pulse time was changed from 5 to 13 s. After that, the gels were stained by ethidium bromide, and the DNA bands were visualized and photographed under a UV transilluminator. Interpretation of chromosomal DNA restriction patterns was performed by visual inspection and based on the criteria proposed by Tenover et al. [13]. Strains showing more than three fragment variations were assumed to represent major PFGE patterns, while those showing one to three fragment differences were considered to represent PFGE pattern subtypes.

### 2.2. HAI Surveillance System in the ICU

Data on ICU patients were retrieved from the active HAI surveillance system that has been conducted on the ward since May 2016 by the Department of Public Health and Infectious Diseases [14]. The ICU is divided into five rooms of two beds each, one large seven-bed room, and one room for patient isolation.

The detailed methodology of the surveillance system was described elsewhere [14]. Briefly, HAIs were diagnosed by an infectious disease specialist, who used a combination of imaging, clinical, and laboratory criteria as defined by the surveillance system’s protocol, which is derived from the National Healthcare Safety Network manual of the Centre for Disease Control [15] and the European Centre for Disease Prevention and Control [16]. All patients hospitalized in the ICU for at least two consecutive calendar days were monitored until their discharge. We collected data on the incidence of (i) catheter-related bloodstream infections (CRBSIs); (ii) ventilation-associated pneumonia (VAP); and (iii) catheter-associated urinary tract infections (CAUTIs) that occurred at least 48 h after device insertion. We also monitored the incidence of BSI of unknown origin (BUO), healthcare-associated pneumonia, and surgical site infections (SSIs) that occurred 48 h after ICU admission or, in case of SSIs, within 30 days of surgery. The presence of microorganisms on the skin, on mucous membranes, or in open wounds, excretions, or secretions without any adverse clinical sign or symptom was considered as colonization, according to the definition proposed by Horan T.C. et al. [17].

Data were collected systematically using a standardized form with four sections: (1) patient demographics and information on hospitalization (date of ICU admission, discharge date, status of the patient at discharge, preexisting comorbidities, Simplified Acute Physiology Score (SAPS) II); (2) exposure to invasive devices (start and end date of the patient’s exposure to urinary catheterization, central venous catheterization, and mechanical ventilation); (3) antibiotic therapy (drug(s) used, start and end date of antibiotic therapy); (4) diagnosed HAIs and microbiological cultures performed (site of infection, dates of HAI onset and microbiological confirmation).

### 2.3. Statistical Analysis

Antibiotic agents belonging to the same class were grouped (Supplementary Table S1). Exposure to any antibiotic class (yes vs. no) was defined as systemic administration (i.e., enteral or parenteral) for at least two consecutive days of at least one antibiotic agent within each class. The ICU mortality rate and the associated Poisson 95% confidence interval (CI) was calculated for 1000 patient-days. Descriptive statistics were obtained using median and interquartile range (IQR) or mean and standard deviation (SD) for continuous variables and proportions for dichotomous and categorical variables. For the univariable analysis, the Wilcoxon rank-sum test was used to compare continuous variables between patients with and without *A. baumannii* isolation, whereas Pearson’s chi-squared test or Fisher’s exact test was used for dichotomous and categorical variables.

A multivariable logistic regression model was built to identify risk factors for *A. baumannii* colonization or infection. The presence of preexisting comorbidities was collapsed into one variable with two modalities (i.e., having at least one chronic condition vs. having none). Variables were included in the model based on expert opinion. Since days of central venous catheterization, days of urinary catheterization, and days of mechanical ventilation were multicollinear (variance inflation factor > 5), only the latter was retained for further analyses. The Hosmer–Lemeshow test was used to evaluate the goodness of fit of the model. As a result, the final model included the following variables: sex (0 = woman; 1 = man); age (continuous); preexisting comorbidity (0 = no, 1 = yes); SAPS II score (continuous); days of mechanical ventilation (continuous); administration of carbapenems, extended-spectrum cephalosporins, glycopeptides, macrolides, oxazolidinones, and/or penicillins until first *A. baumannii* isolation for the first cohort or until the end of hospitalization for the second cohort (0 = no, 1 = yes). Adjusted odds ratios (aORs) and 95% CIs were calculated.

A sensitivity analysis was performed, distinguishing patients who were primarily colonized or infected by *A. baumannii*. In particular, we used an extension of the binomial logistic regression model, i.e., the multinomial logistic regression model, to simultaneously compare three groups: patients with no *A. baumannii* acquisition, patients who were primarily colonized, and patients who were primarily infected. The same variables used in the logistic regression model were used. Adjusted relative risk ratio (aRRR) and 95% CIs were calculated. All analyses were performed using Stata (StataCorp LLC, 4905 Lakeway Drive, College Station, TX, USA), version 17.0. A two-sided *p*-value < 0.05 was considered statistically significant.

## 3. Results

### 3.1. Molecular Typing and Characteristics of A. baumannii Isolates

**Figure 1:**
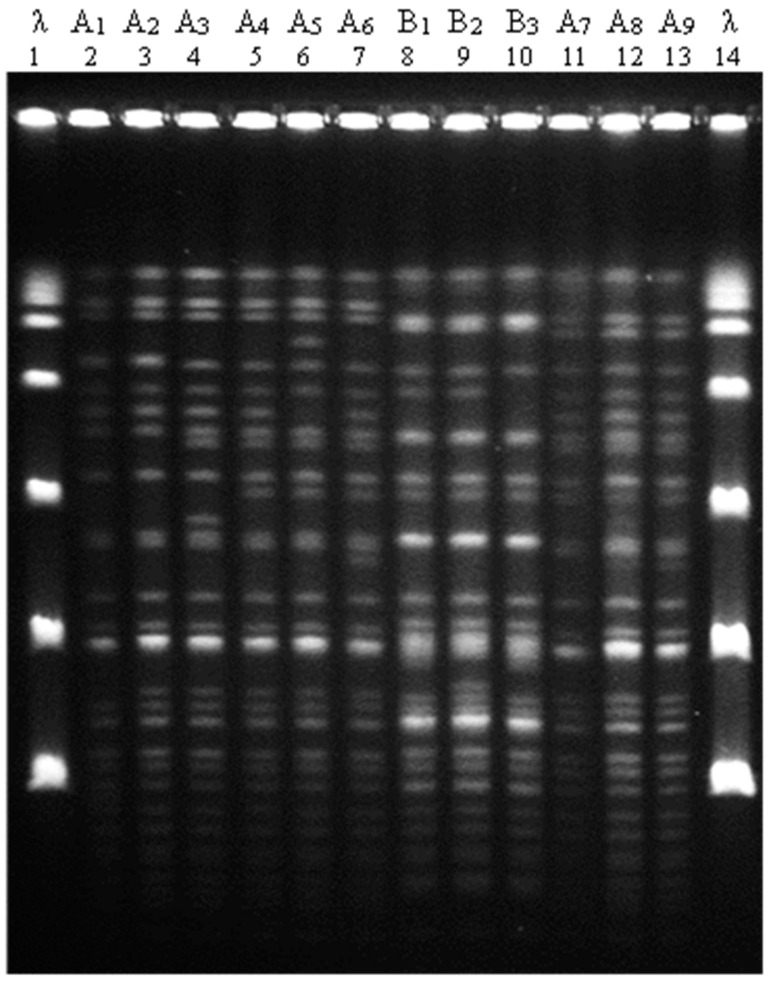
Apa I PFGE patterns of *A. baumannii* strains isolated from SARS-CoV-2 patients admitted to the intensive care unit of the Umberto I teaching hospital of Rome between 1 March 2020 and 28 February 2021. Representative isolates of *A. baumannii* strains are shown in lanes 2–7, 11–13 (PFGE pattern A), and 8–10 (pattern B). Lines 1 and 14 contain molecular size patterns (lambda ladder 48.5 kb).

During the one-year study period, 193 SARS-CoV-2 patients were admitted to the ICU of the Umberto I teaching hospital, from whom 147 strains of *A. baumannii* were isolated. Of these, 102 strains (69.4%) belonging to 59 patients (average per patient: 1.7 ± 0.7 isolates) were genotyped by macrorestriction chromosomal DNA analysis and PFGE. The analysis showed two major PFGE patterns, which we named A and B and further classified into subtypes: A_1_ (1 strain), A_2_ (1 strain), A_3_ (2 strains), A_4_ (27 strains), A_5_ (2 strain), A_6_ (3 strains), A_7_ (12 strains), A_8_ (1 strain), A_9_ (3 strains), B_1_ (46 strains), B_2_ (2 strains), and B_3_ (2 strains) (Figure 1). Considering the 33 patients who were interested by multiple *A. baumannii* isolates, 26 were colonized and/or infected by strains with the same genotype pattern, while only 7 were found with isolates presenting different PFGE subtypes.

**Figure 2:**
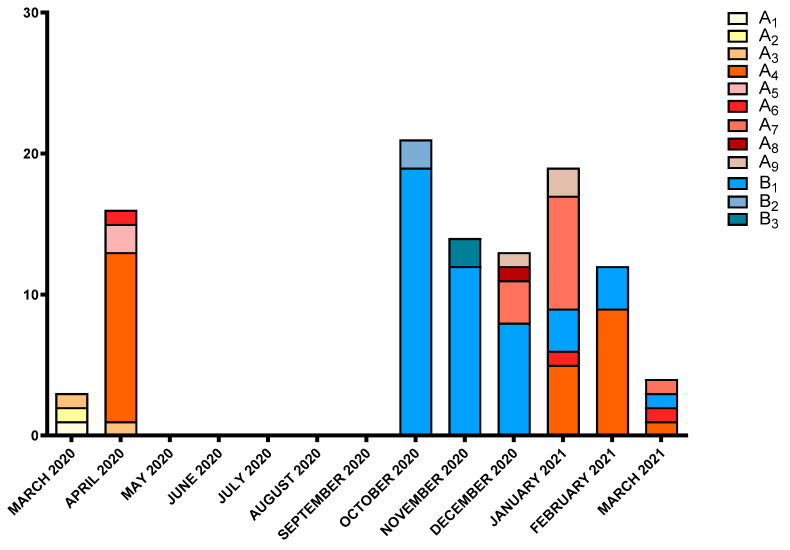
Temporal distribution and frequency of the PFGE patterns of *A. baumannii* strains isolated from SARS-CoV-2 patients admitted to the intensive care unit of Umberto I teaching hospital between 1 March 2020 and 28 February 2021 (follow-up extended until 30 March 2021).

The most prevalent PFGE subtypes occurred in a well-defined temporal period: subtype A_4_ (26.5%) was predominant in April 2020 and then disappeared before showing up again in January–March 2021; subtype A_7_ (11.8%) was found between December 2020 and January 2021 and again in March 2021. Subtype B_1_ (45.1%), however, appeared in October 2020, and its prevalence decreased in the following months (Figure 2).

Around 40% of the genotyped isolates were responsible for HAIs: the majority were VAP for both genotypes patterns (pattern A: *n* = 18 and pattern B: *n* = 10), followed by CAUTIs (pattern A: *n* = 2 and pattern B: *n* = 4) (Table 1). The remaining genotyped strains were colonizations, in which the bacterium was isolated mainly from rectal swab (38.5% of pattern A and 36.0% of pattern B), followed by bronchial aspirate (15.4% of pattern A and 2.0% of pattern B), central venous catheter and urine (4.0% each in pattern B only), and abdominal drainage (2.0% of pattern A). As for the room of detection, the vast majority of isolates belonging to pattern A were found in the large seven-bed room (63.5%), whereas the distribution of pattern B strains was more balanced between the large room (26.0%), room 4 (22.0%), and room 5 (20.0%).

Lastly, regarding the resistance profiles of these bacteria, all *A. baumannii* isolates were resistant to gentamicin (*n* = 3 had minimum inhibitory concentration [MIC] > 8, and *n* = 99 had MIC ≥ 16), meropenem (MIC ≥ 16), imipenem (MIC ≥ 16), and ciprofloxacin (*n* = 3 had MIC > 2, and *n* = 99 had MIC ≥ 4), while they were all susceptible to colistin (*n* = 3 had MIC ≤ 2 and *n* = 99 had MIC ≤ 0.5) (data not shown). Interestingly, while pattern B strains were mostly susceptible to trimetoprim/sulfametoxazolo (90.0% had MIC ≤ 20) or showed intermediate resistance (6.0% had MIC = 80), pattern A isolates were all resistant (MIC ≥ 320).

### 3.2. ICU Nosocomial Infections

During the one-year study period, 193 patients were admitted to the ICU (45 patients from March to August 2020 and 148 patients from September 2020 to February 2021). There were 136 men (70.5%) and 57 women (29.5%) aged 63.2 years on average (SD: 13.5 years). The 193 patients spent a total of 3017 days in the ICU (average = 15.6 ± 11.8 days), with device utilization ratios of 0.97, 0.74, and 0.83 for urinary catheter, central line, and invasive ventilation, respectively. The overall mortality incidence was 62.2% (120 of 193). A total of 169 infections were detected among 86 patients, for an overall nosocomial infection incidence of 44.6%. The most common infections were CAUTIs (38.5%), VAP (37.3%), and BUO (21.9%), with only four CRBSIs (2.4%).

Five pathogens (*A. baumannii*, *Klebsiella pneumoniae*, *Pseudomonas aeruginosa*, *Candida* spp., *Staphylococcus aureus*, and *Enterococcus faecium*) were responsible for more than 70% of the infections that occurred in the unit during the study period. More precisely, *A. baumannii* and *Candida* spp. caused 52 (30.8%) and 31 (18.3%) infections, whereas *K. pneumoniae*, *P. aeruginosa*, *S. aureus*, and *E. faecium* were responsible for 18 (10.7%), 14 (8.3%), 5 (3.0%), and 5 (3.0%) infections, respectively. Other less-frequently isolated pathogens included *Enterococcus* spp., *Enterobacter* spp., *Escherichia coli*, *Klebsiella* spp., *Proteus mirabilis*, coagulase-negative *staphylococci*, and *Stenotrophomonas maltophilia*.

### 3.3. Risk Factors for A. baumannii Acquisition

The cumulative incidence of *A. baumannii* acquisition (colonization or infection) was 36.8% (Table 2). Among the 71 patients in which *A. baumannii* was found, the first isolate was collected after a median of 12 days (IQR: 7–17 days). Comparing patients with and without *A. baumannii* isolation, most patients were male in both groups (67.6% and 72.1%), with a similar median age (63 vs. 65 years). Hypertension was the most prevalent comorbidity in both cohorts (45.1% and 43.4%), followed by diabetes mellitus (16.9% and 18.9%), cancer (9.9% and 10.7%), chronic obstructive pulmonary disease (8.5% and 9.8%), and cardiovascular disease (11.3% and 8.2%). Only chronic kidney disease seemed to be slightly more frequent among patients without *A. baumannii* isolation (9.8% vs. 1.4%). The median SAPS II score at ICU admission was comparable (33 vs. 35 points). In the group of patients with *A. baumannii* isolation, at the end of a median length of stay of 22 days (IQR: 13–28), 52 patients had died (73.2%), accounting for an ICU mortality rate of 31.4 per 1000 patient-days. By contrast, in the second group, after a shorter follow-up time (median: 9 days, IQR: 6–14), 68 patients had died (55.7%), for a corresponding ICU mortality rate of 49.9 per 1000 patient-days. Overall, the median use of urinary catheter, central line and mechanical ventilation was more than twice as high in the former than in the latter cohort (22 vs. 8.5 days, 16 vs. 5 days, and 17 vs. 6 days, respectively). Lastly, previous antibiotic consumption was high in both groups; patients with *A. baumannii* had higher use of carbapenems than patients without isolation of the pathogen (47.9% vs. 24.6%), while they showed comparable use of other antibiotics.

In the multivariable analysis, higher odds of *A. baumannii* acquisition were found for previous consumption of carbapenems only (aOR: 4.15, 95% CI: 1.78–9.64) (Table 3). Age, sex, preexisting comorbidity, SAPS II score, mechanical ventilation, and previous consumption of other antibiotic classes did not seem to be risk factors for the outcome.

Sensitivity analysis showed similar results (Supplementary Table S2). Specifically, previous consumption of carbapenems was associated with an increased risk of both colonization and infection by *A. baumannii* (aRRR: 4.36, 95% CI: 1.67–11.39 and aRRR: 4.05, 95% CI: 1.24–13.20, respectively), whereas none of the other factors seemed to be related to any event.

## 4. Discussion

Given the increase in the number of infections caused by *A. baumannii* and the emergence of MDR and XDR strains in recent years [18], this pathogen has become an increasing concern for ICU patients [19] who, because they frequently have critical conditions, may be particularly vulnerable to infections or, in the case of COVID-19 patients, coinfections [20]. In this study, all isolates analysed showed a similar multidrug-resistant antibiotype, leading us to hypothesize a certain degree of clonality between them. The presence of high levels of cross-contamination on the ward was confirmed by the discovery of two main patterns and a few subgroups, as shown by PFGE analysis. In addition, since isolates belonging to the same pulsotype were collected not only in different patients hospitalized within the same period but in patients who had not shared any time together in the ICU, it is likely that some transmission events occurred indirectly via the contaminated environment and healthcare personnel, similarly to what has been observed by other authors [21,22]. In this regard, it is well known that inadequate application of preventive practices promotes cross-contamination, allowing microorganisms such as *A. baumannii* to colonize hospital environments and persist for long periods [23]. During the COVID-19 pandemic, given that contact precautions designed to minimize the transmission risk were based on the COVID-19 status, and not the MDR microorganism (MDRO) carrier status, of patients [24], efforts to minimize the spread of the virus may have led to gaps in routine infection control activities, thereby facilitating the transmission of other infectious agents [22]. Indeed, while before the COVID-19 pandemic, acquisition of *A. baumannii* was described after a mean hospital stay of 42 days [24], we observed a much shorter time window, in line with Gottesman et al. [24]. Moreover, we collected the greatest number of isolates from the large room in the ICU, probably because of the higher number of occupied beds in this space, but also because the longer residence time of COVID-19 patients may have created fewer opportunities for terminal cleaning, especially in multipatient rooms [24]. Hence, as the pandemic continues, understanding transmission routes and applying containment strategies, including routine environmental cleaning and disinfection, could help hospitals to prevent clusters and respond promptly when they are detected [9].

In line with the literature [8], our COVID-19 patients required intense levels of support, with prolonged exposure to invasive devices coupled with high mortality rates, both indications of their severe clinical condition [25,26]. Furthermore, almost half the patients developed at least one infection during their ICU stay, a third of which were sustained by *A. baumannii.* Thus, the burden of this microorganism was particularly consistent in our ICU, and given its multidrug resistance profile and the limited treatment options [27], it deserved special attention. For this reason, and since both colonization and infection in patients are important sources of the spread of resistant strains in hospital settings [28], we investigated the characteristics of patients with and without *A. baumannii*, regardless of whether they were infected or colonized. While the lower mortality rate in patients with the bacterium probably reflected their prolonged hospital stays (more than twice that of patients without *A. baumannii*), a higher proportion of these patients died. These findings align with the literature [19], in which *A. baumannii* acquisition has been associated with both hospital residence time and ICU mortality. Hence, given the serious implications for the clinical outcomes of these patients [29], the isolation of this bacterium from COVID-19 patients underscores the importance of appropriate prevention and control practices [30,31,32]. Evidence-based interventions should be conducted to improve the awareness of and adherence to hygiene precautions in healthcare workers and to limit clonal transmission as much as possible [32,33].

Among the factors that may play a role in acquisition of *A. baumannii*, variables relating to the demographic characteristics of patients (e.g., sex, age) or their clinical conditions (e.g., SAPS II, comorbidities, use of invasive devices) have been found to increase the risk, albeit not consistently [34,35,36,37]. However, the availability of data on the issue among COVID-19 patients is still scarce [38]. In our study, the presence of *A. baumannii* seemed to be related to patient treatment in both main and sensitivity analysis. As hypothesized in other studies [39,40,41], carbapenems are broad-spectrum antibiotics with activities against most Gram-negative bacteria; therefore, it is possible that using these antibiotics could change the patients’ bacterial flora and facilitate the colonization and/or infection of resistant bacteria, including *A. baumannii* [42]. In this regard, it is known that COVID-19 patients experience high rates of antibiotic use all over the world [43,44], and our patients were no exception. Since antibiotic use has been recognized as a major cause of antimicrobial resistance [45,46], limiting their use is critical in reducing the emergence and spread of MDR bacteria such as *A. baumannii* [47]. However, it is important to remember that other microorganisms were circulating in the ICU during the same period, and that carbapenems are clinically useful and sometimes even the last-resort drugs against some microorganisms in current clinical practice [48]. Therefore, implementing stewardship programs to rationalize the use of such antibiotics could help to reduce the selective pressures that favour highly resistant pathogens, including *A. baumannii* [49].

This study had several strengths and limitations. The main strength was that data on patients and nosocomial infections were collected as part of a four-year surveillance system that was routinely used in the ICU, meaning that potential bias in results due to overloading of ward staff was unlikely. In addition, we were able to perform molecular typing in a consistent proportion of the *A. baumannii* isolates (~70%), which allowed us to confirm a certain degree of clonal transmission. By contrast, the first limitation was the fact that since, during the pandemic, entry into the ICU was limited to ward staff only, environmental surveillance activities were not allowed, and we could not investigate the clonal relationship between clinical and environmental strains of *A. baumannii*. Second, the interpretation of PFGE results was performed by visual inspection following Tenover’s criteria [13]. The use of specialized software may facilitate genotypic comparison, but it usually returns results similar to those obtained using Tenover’s guidelines [50]. Therefore, we believe that using such criteria may have been an adequate method to investigate the clonal transmission in a hospital environment limited to a single unit. Third, we used *A. baumannii* acquisition, including colonization and infection, as the main outcome. For this reason, the study results may have differed from those of studies focusing only on infection. However, we think that including both colonization and infection made our findings relevant for infection control interventions, because colonization usually precedes infection in most patients, and patients with *A. baumannii* colonization are important sources of cross-transmission. In addition, we performed a sensitivity analysis differentiating patients who were primarily colonized and infected, and no substantial change was observed in relation to risk factors, meaning that the overall meaning of our findings was preserved. Another limitation was that as soon as patients were discharged from the ICU, they were no longer under surveillance. However, only the most stable were chosen for transfer. Lastly, even though it was not the goal of our study, we did not perform a multivariable analysis of the clinical impact of the presence of *A. baumannii* in infected patients. Similarly, we did not investigate the genes responsible for the observed resistance to carbapenems, but they are an interesting area for future research.

## 5. Conclusions

Because of its high degree of prevalence and multidrug resistance to antibiotics, we found that *A. baumannii* may represent a major threat to COVID-19 patients. In addition, the high levels of clonal transmission that we documented indicate that effective prevention and containment strategies should be implemented as soon as possible. These include, but are not limited to, contact precautions for *A. baumannii* carriers, meticulous environmental cleaning and disinfection, and tailored interventions to promote antimicrobial stewardship, as well as awareness of and adherence to hygiene guidelines among healthcare workers [24].

## Figures and Tables

**Table 1:** Characteristics of the genotyped *A. baumannii* strains isolated by major pulsed-field gel electrophoresis (PFGE) pattern from SARS-CoV-2 patients admitted to the intensive care unit of the Umberto I teaching hospital of Rome between 1 March 2020 and 28 February 2021. Results are expressed as number and percentage.

			PFGE Pattern A	PFGE Pattern B
			*n* (%)	*n* (%)
Isolate type				
	Healthcare-associated infection			
		VAP	18 (34.6)	10 (20.0)
		CAUTI	2 (3.8)	4 (8.0)
		BUO	2 (3.8)	2 (4.0)
		CRBSI	1 (1.9)	2 (4.0)
	Colonization			
		Bronchial aspirate	8 (15.4)	10 (20.0)
		Central venous catheter	0 (0.0)	2 (4.0)
		Urine	0 (0.0)	2 (4.0)
		Abdominal drainage	1 (2.0)	0 (0.0)
		Rectal swab	20 (38.5)	18 (36.0)
Room of detection				
	Room 1		1 (1.9)	7 (14.0)
	Room 2		0 (0.0)	9 (18.0)
	Room 3		4 (7.7)	0 (0.0)
	Room 4		4 (7.7)	11 (22.0)
	Room 5		5 (9.6)	10 (20.0)
	Isolation room		5 (9.6)	0 (0.0)
	Large Room		33 (63.5)	13 (26.0)

VAP: ventilator-associated pneumonia. CAUTI: catheter-associated urinary tract infection. CRBSI: catheter-related blood stream infection. BUO: bloodstream infection of unknown origin.

**Table 2:** Characteristics of the SARS-CoV-2 patients admitted to the intensive care unit of the Umberto I teaching hospital of Rome between 1 March 2020 and 28 February 2021. Results are expressed as number (percentage) or median (interquartile range).

		Colonization or Infection by *A. baumannii*		
		Yes	No	*p*-Value
Patients		71	122	
Observation time, person-days		1654	1363	
Age (years)		63 (54–71)	65 (57–74)	0.220
Gender				0.506
	Male	48 (67.6)	88 (72.1)	
	Female	23 (32.4)	34 (27.9)	
Preexisting comorbidity				
	Hypertension	32 (45.1)	53 (43.4)	0.957
	Diabetes mellitus	12 (16.9)	23 (18.9)	0.734
	Cancer	7 (9.9)	13 (10.7)	0.861
	Chronic obstructive pulmonary disease	6 (8.5)	12 (9.8)	0.750
	Cardiovascular disease	8 (11.3)	10 (8.2)	0.479
	Chronic liver failure	0 (0.0)	1 (0.8)	0.999
	Chronic kidney failure	1 (1.4)	12 (9.8)	0.034
	Neutropenia	0 (0.0)	3 (2.5)	0.299
	Transplant	0 (0.0)	1 (0.8)	0.999
	Asthma	4 (5.6)	3 (2.5)	0.264
	Bronchiectasis	0 (0.0)	1 (0.8)	0.999
SAPS II score		33 (26–39)	35 (28–43)	0.176
ICU deaths		52 (73.2)	68 (55.7)	0.016
Mortality rate (95% CI) per 1000 patient-days		31.4 (23.4–41.2)	49.9 (38.7–63.2)	0.012
Total length of ICU stay, days		22 (13–28)	9 (6–14)	<0.001
Total use of urinary catheter, days		22 (13–28)	8.5 (6–14)	<0.001
Total use of central venous catheter, days		16 (6–24)	5 (0–10)	<0.001
Total use of mechanical ventilation, days		17 (6–24)	6 (2–10)	<0.001
Antibiotic consumption *				
	Carbapenems	34 (47.9)	30 (24.6)	<0.001
	Extended-spectrum cephalosporins	14 (19.7)	21 (17.2)	0.663
	Glycopeptides	39 (54.9)	67 (54.9)	0.999
	Macrolides	41 (57.8)	59 (48.4)	0.208
	Oxazolidinones	10 (14.1)	10 (8.2)	0.196
	Penicillins	41 (57.8)	79 (64.8)	0.333

* Antibiotic consumption was calculated until first *A. baumannii* isolation (cohort I) or the end of hospitalization (cohort II). CI: confidence interval; SAPS: Simplified Acute Physiology Score.

**Table 3:** Multivariable logistic regression model for colonization or infection by *A. baumannii* among the SARS-CoV-2 patients admitted to the main intensive care unit of the Umberto I teaching hospital of Rome between 1 March 2020 and 28 February 2021.

	Colonization or Infection by *A. baumannii*	
	OR (95% CI)	*p*-Value
Age (years)	1.00 (0.97–1.03)	0.865
Sex (male)	1.20 (0.60–2.39)	0.611
Preexisting comorbidity (yes)	0.87 (0.44–1.72)	0.691
SAPS II score	0.98 (0.94–1.01)	0.181
Mechanical ventilation, days	1.01 (0.97–1.05)	0.643
Previous consumption of carbapenems (yes)	4.15 (1.78–9.64)	0.001
Previous consumption of extended-spectrum cephalosporins (yes)	1.03 (0.43–2.45)	0.942
Previous consumption of glycopeptides (yes)	0.82 (0.39–1.71)	0.591
Previous consumption of macrolides (yes)	1.80 (0.93–3.51)	0.083
Previous consumption of oxazolidinones (yes)	0.91 (0.29–2.82)	0.872
Previous consumption of penicillins (yes)	1.02 (0.49–2.12)	0.960

OR: odds ratio; CI: confidence interval; SAPS: Simplified Acute Physiology Score.

## Data Availability

Data available on reasonable request because of privacy reasons.

## Supplementary Materials

The following supporting information can be downloaded at: https://www.mdpi.com/article/10.3390/microorganisms10040722/s1, Table S1: Classification of antibiotic agents into their respective antibiotic classes. Table S2: Multinomial logistic regression model for *A. baumannii* colonization or infection among SARS-CoV-2 patients admitted to the intensive care unit of the Umberto I teaching hospital of Rome between 1 March 2020 and 28 February 2021.
