# Supplementary material for: Acinetobacter baumannii Isolates from COVID-19 Patients in a Hospital Intensive Care Unit: Molecular Typing and Risk Factors
Source: Microorganisms. 2022 Mar 28;10(4):722. doi: 10.3390/microorganisms10040722 (PMC9026468; doi:10.3390/microorganisms10040722)
Supplement: Supplementary file 1 [file microorganisms-10-00722-s001.zip › microorganisms-1592759-supplementary.pdf]

Supplementary Table S1. Classification of antibiotic agents into their respective antibiotic class.

Antibiotic class	Antibiotic agent
Aminoglycosides	Gentamicin
	Amikacin
	Paromomycin
Carbapenems	Ertapenem
	Imipenem
	Meropenem
Extended spectrum cephalosporines	Cefotaxime
	Ceftazidime
	Cefepime
	Ceftriaxone
	Ceftazidime/Avibactam
	Cefoperazone
	Ceftolozane/Tazobactam
Glycopeptides	Vancomycin
	Teicoplanin
Daptomycin	Daptomycin
Linezolid	Linezolid
Penicillin	Amoxicillin/Clavulanic acid
	Ampicillin/Sulbactam
	Piperacillin/Tazobactam
Colistin	Colistin

Supplementary Table S2. Multinomial logistic regression model for *A. baumannii* colonization or infection among SARS-CoV-2 patients admitted to the intensive care unit of the Umberto I teaching hospital of Rome between 1 March 2020 and 28 February 2021.

	**Colonization by *A. baumannii***		**Infection by *A. baumannii***	
	**RRR (95% CI)**	***p*-Value**	**RRR (95% CI)**	***p*-Value**
Age (years)	0.99 (0.96-1.02)	0.399	1.03 (0.98-1.07)	0.246
Sex (male)	1.02 (0.45-2.30)	0.959	1.72 (0.64-4.60)	0.283
Pre-existing comorbidity (yes)	1.00 (0.46-2.20)	0.993	0.60 (0.22-1.62)	0.311
SAPS II score	1.00 (0.96-1.04)	0.845	0.93 (0.87-0.99)	0.016
Mechanical ventilation, days	1.00 (0.95-1.05)	0.929	1.04 (0.98-1.10)	0.213
Previous consumption of carbapenems (yes)	4.36 (1.67-11.39)	0.003	4.05 (1.24-13.20)	0.020
Previous consumption of extended spectrum cephalosporins (yes)	1.46 (0.57-3.72)	0.430	0.36 (0.07-1.81)	0.215
Previous consumption of glycopeptides (yes)	0.69 (0.29-1.63)	0.403	1.17 (0.39-3.51)	0.780
Previous consumption of macrolides (yes)	1.97 (0.92-4.23)	0.080	1.49 (0.55-4.01)	0.429
Previous consumption of oxazolidinones (yes)	0.60 (0.16-2.34)	0.465	1.93 (0.44-8.50)	0.385
Previous consumption of penicillins (yes)	1.25 (0.54-2.86)	0.605	0.66 (0.22-1.96)	0.453

RRR: Relative Risk Ratio. CI: Confidence Interval. SAPS: Simplified Acute Physiology Score.
